# Review of public motor imagery and execution datasets in brain-computer interfaces

**DOI:** 10.3389/fnhum.2023.1134869

**Published:** 2023-03-30

**Authors:** Daeun Gwon, Kyungho Won, Minseok Song, Chang S. Nam, Sung Chan Jun, Minkyu Ahn

**Affiliations:** ^1^Department of Computer Science and Electrical Engineering, Handong Global University, Pohang, Republic of Korea; ^2^School of Electrical Engineering and Computer Science, Gwangju Institute of Science and Technology, Gwangju, Republic of Korea; ^3^Edward P. Fitts Department of Industrial and Systems Engineering, North Carolina State University, Raleigh, NC, United States; ^4^Department of Industrial and Management Systems Engineering, Kyung Hee University, Yongin-si, Republic of Korea; ^5^AI Graudate School, Gwangju Institute of Science and Technology, Gwangju, Republic of Korea; ^6^School of Computer Science and Electrical Engineering, Handong Global University, Pohang, Republic of Korea

**Keywords:** brain-computer interface (BCI), motor imagery, motor execution, public dataset, data quality, meta-analysis

## Abstract

The demand for public datasets has increased as data-driven methodologies have been introduced in the field of brain-computer interfaces (BCIs). Indeed, many BCI datasets are available in various platforms or repositories on the web, and the studies that have employed these datasets appear to be increasing. Motor imagery is one of the significant control paradigms in the BCI field, and many datasets related to motor tasks are open to the public already. However, to the best of our knowledge, these studies have yet to investigate and evaluate the datasets, although data quality is essential for reliable results and the design of subject− or system-independent BCIs. In this study, we conducted a thorough investigation of motor imagery/execution EEG datasets recorded from healthy participants published over the past 13 years. The 25 datasets were collected from six repositories and subjected to a meta-analysis. In particular, we reviewed the specifications of the recording settings and experimental design, and evaluated the data quality measured by classification accuracy from standard algorithms such as Common Spatial Pattern (CSP) and Linear Discriminant Analysis (LDA) for comparison and compatibility across the datasets. As a result, we found that various stimulation types, such as text, figure, or arrow, were used to instruct subjects what to imagine and the length of each trial also differed, ranging from 2.5 to 29 s with a mean of 9.8 s. Typically, each trial consisted of multiple sections: pre-rest (2.38 s), imagination ready (1.64 s), imagination (4.26 s, ranging from 1 to 10 s), the post-rest (3.38 s). In a meta-analysis of the total of 861 sessions from all datasets, the mean classification accuracy of the two-class (left-hand vs. right-hand motor imagery) problem was 66.53%, and the population of the BCI poor performers, those who are unable to reach proficiency in using a BCI system, was 36.27% according to the estimated accuracy distribution. Further, we analyzed the CSP features and found that each dataset forms a cluster, and some datasets overlap in the feature space, indicating a greater similarity among them. Finally, we checked the minimal essential information (continuous signals, event type/latency, and channel information) that should be included in the datasets for convenient use, and found that only 71% of the datasets met those criteria. Our attempts to evaluate and compare the public datasets are timely, and these results will contribute to understanding the dataset’s quality and recording settings as well as the use of using public datasets for future work on BCIs.

## 1. Introduction

Electroencephalography (EEG) signals interact by reflecting an individual’s real-time state, so they can be used to predict and classify emotions, attention, and imagination. A brain-computer interface (BCI) uses EEG for applications in active, reactive, and passive manners as needed (Gürkök and Nijholt, 2012). Reactive BCIs, such as P300 based upon event-related potential (ERP) (Guy et al., 2018) and steady-state visual evoked potential (SSVEP) (Yin et al., 2015), manipulate applications by utilizing EEG responses to stimuli. Motor imagery (MI) BCI uses the brainwave pattern that occurs during an intrinsic rehearsal of movement and is an interface method that can be controlled intuitively with active BCIs (Annett, 1995).

Motor imagery (MI) is responsible for the cognitive processes of motor behavior and shares neural mechanisms with actual movements. In fact, in both imaginary and actual movements (or motor execution, ME), the neural activation of event-related EEG in the Mu rhythm (8−12 Hz) of the motor cortex was observed as being functionally similar (Llanos et al., 2013). Although there was a difference in the intensity of brain activities during MI and ME, in a previous study that MEG, the event-related synchronization/desynchronization (ERS/ERD) of beta (15−30 Hz) in the contralateral motor cortex and somatosensory cortex has been confirmed commonly (Kraeutner et al., 2014). Based on these neurophysiological characteristics, MI-BCI is used for the rehabilitation of brain functions in patients with motor disorders. MI offers additional advantages to conventional physiotherapy or occupational therapies during the rehabilitation of movement disorders in stroke patients (Zimmermann-Schlatter et al., 2008). In addition, the possibility of using MI in the rehabilitation process of diseases that cause movement disorders, such as patients with cerebral palsy (Steenbergen et al., 2009) and Parkinson’s disease (Caligiore et al., 2017), has been suggested.

However, MI-based BCIs have a challenge occasionally, referred to “BCI illiteracy phenomenon” in which some potential users may not reach a sufficient performance level to control the BCI applications (Blankertz et al., 2010). They can be considered prospective users, as their poor performance may be attributable to external factors such as the BCI training protocol rather than personal characteristics (Thompson, 2019). Approximately 20% of BCI users have been considered BCI illiterate, with a performance unsuitable for using BCI applications (Edlinger et al., 2015). Imagination-based BCI paradigms have lower accuracy than reactive or passive paradigms (Gürkök and Nijholt, 2012; Lee et al., 2019). In fact, in an MI-based BCI experiment with 80 BCI beginners, it was reported that approximately 40% were classified as BCI poor performers (Sannelli et al., 2019).

High-quality EEG signals requires a controlled environment, long-term recordings for calibration sessions, and expensive equipment for reliable operation (Pfurtscheller et al., 2000; Thomas et al., 2013). Further, unlike the reactive paradigms, the MI task imposes a high workload and consequently fatigues subjects because they have to imagine movement while suppressing actual movement. These fatigue levels have been found to be related to MI performance (Talukdar et al., 2019). Therefore, long-term measurements in a single experiment can degrade the data quality, so the cost of measurement data is high because it takes much time for the experimenter to obtain a large amount of MI data.

Recently, research to improve BCI performance by using machine learning and deep learning has been conducted actively (Roy et al., 2019). For stable model learning, it is essential to have a sufficient number of data and high-quality data (Jain et al., 2020). Therefore, there is a high demand for public data among BCI researchers. The data related to the movement paradigms, MI and ME, are being provided through various platforms, such as *MOABB* (Jayaram and Barachant, 2018), *BNCI Horizon* (Brunner et al., 2015), and *Deep BCI* (Deep BCI, n.d.).

Although the accessibility of public data has increased, BCI-competitive datasets the Berlin BCI (BBCI) group released in 2008 are still used the most widely (Tangermann et al., 2012; Al-Saegh et al., 2021; Alzahab et al., 2021). There may be several reasons why researchers still use a small dataset consisting of nine subjects although larger datasets have been made available to the public recently. First, researchers want to substantiate their research results through sufficiently verified data. Therefore, they prefer to use a dataset that compares results with those of previous studies. Second, the reason why a new dataset is not used in addition to a conventional dataset may be attributable to a compatibility problem between the different datasets. The EEG signal depends on the subjects because of its non-stationary nature, and the environment affects it greatly because of its sensitivity to noise (Kaplan et al., 2005). Therefore, most of the studies that have used public datasets were evaluating the model’s performance by building independent models for each dataset to validate the model (Luciw et al., 2014; Miao et al., 2017; Tang et al., 2019; Tayeb et al., 2019). Finally, researchers may not be informed sufficiently about datasets published recently.

Experimenters can even configure the same paradigm in various ways, such as by inter-trial-intervals (ITI) and stimuli presentation (Sarma and Barma, 2020). The SSVEP and P300 paradigm parameters have been considered to have a significant effect on performance and much research has been conducted on them (Gonsalvez and Polich, 2002; Zhu et al., 2010; Han et al., 2022). However, in MI BCI, a similar cue-based experiment has been used continuously in the past (Pfurtscheller and Neuper, 1997) and in recent studies (Tibrewal et al., 2022), although MI parameters may also influence signal quality or BCI performance. Indeed, few studies have attempted to investigate the MI paradigm’s effect.

This study reviews movement datasets on the MI or ME paradigms collected from various resources, such as journals, research projects, and platforms. The ME datasets were also included in the study so that researchers can refer to them to find the potential biomarkers of motor function. Many EEG public data for motor tasks have been shared, but the data’s quality needs to be assessed. Accordingly, we evaluated usability, paradigm parameters, classification accuracy, and compatibility with other datasets. To use the data, sufficient information must be provided to the user. Comparing the specifications of the dataset, we provide information so that researchers can select the dataset desired according to the purpose (e.g., paradigm) or condition (e.g., environment). In addition, classification analysis was performed on representative datasets under the same requirements to compare objective classification accuracy, and compatibility between datasets was inferred using the features extracted. Finally, we report the limitations of current public datasets from a practical perspective and suggest the direction of future discussions about public datasets.

## 2. Materials and methods

### 2.1. Public dataset collection and organization

We collected public datasets for motor tasks, including MI and ME, from several resources, including journals *Scientific Data* (Scientific Data, n.d.) and *Gigascience* (GigaScience | Oxford Academic, n.d.), research projects (*Deep BCI* and *BNCI Horizon*), and dataset platforms (*IEEE DataPort* (IEEE DataPort, n.d.) and *MOABB*). The characteristics of each resource are as follows. *Gigascience* and *Scientific Data* are journals that provide the source of the dataset, and *Gigascience* shares the dataset through its database, while *Scientific Data* shares the dataset through such repositories as *Figshare* without requiring a separate license for both. *Deep BCI* and *BNCI Horizon* are research projects that collect shared datasets published in a paper or requested by the projects. Researchers can download data directly from the *BNCI Horizon* website, while *Deep BCI* can receive the dataset after obtaining consent from the data owner. *IEEE Dataport* is a data platform that provides a dataset and simple information, and *MOABB* is a BCI benchmark that offers data and available Python APIs. Among them were datasets released multiple times from other resources and datasets the projects requested.

We collected a total of 25 datasets based on the following criteria:

• Should include EEG.
• Should include healthy subjects.
• Should include datasets that do not overlap with previous data from other resources.
• Should be available currently.
• Should be presented in English.

There were 17 MI datasets that met these criteria (Leeb et al., 2007; Grosse-Wentrup et al., 2009; Faller et al., 2012; Tangermann et al., 2012; Ahn et al., 2013a; Yi et al., 2014; Lee et al., 2016, 2019; Steyrl et al., 2016; Zhou et al., 2016; Cho et al., 2017; Shin et al., 2017; Kim et al., 2018; Ma et al., 2020; Wu, 2020; Zhou, 2020; Stieger et al., 2021), 4 ME datasets (Luciw et al., 2014; Brantley et al., 2018; Wagner et al., 2019; Schwarz et al., 2020), and 4 both MI and ME datasets (Schalk et al., 2004; Ofner et al., 2017; Kaya et al., 2018; Jeong et al., 2020). In this study, the naming of the dataset took the form of representing the first author’s last name and the year of the published paper or dataset. For example, Stieger (2021) indicates the dataset in Stieger et al. (2021).

### 2.2. Classification and feature analysis

We conducted a quantitative analysis of eight representative MI datasets to compare their compatibility: Ahn et al., 2013a; Yi et al., 2014; Cho et al., 2017; Shin et al., 2017; Kaya et al., 2018; Kim et al., 2018; Lee et al., 2019; Stieger et al., 2021. These were selected as comparable according to various conditions, such as device, instruction method, and cue type, and were measured by the four EEG devices used most commonly—Neuroscan SynAmps2, BranProduct BrainAmp, Neurofax EEG-1200, and Biosemi—and included the basic MI paradigm, left- and right-handed imagination. The paradigm of the dataset is similar in the framework of imagination according to the instruction (cue) after rest (fixation cross), but the details differed, such as cue display and imagination methods.

To assess the data quality and compatibility with other datasets, we calculated the classification accuracy of the binary class problems and the feature distance among other datasets. As each dataset has a different number of channels, sampling rate, and signal scale, each dataset needs to be projected onto the same feature space for comparison. We extracted the features using the CSP, which is one of the methods to classify binary MI problems used most commonly (Aggarwal and Chugh, 2019). Our goal was to quantify it as objectively as possible by analyzing different datasets comparatively under common conditions. The CSP constructs a spatial filter to maximize one class and minimize the other class on the binary conditions (Ramoser et al., 2000).

The time-series data that have undergone a series of pre-processing processes extracts the CSP features through the following process. Let *x*_*i*_ ∈ *R*^*N*×*S*^ be the pre-processed EEG signal that consists of channels *N* -by-sample *S* as a multi-channel time series for a particular class motor imagery *i* such as the left- and right-hand. The spatial covariance matrix is *C^i^* ∈ *R*^*N*×*N*^, given in Eq. 1:


(1) $$C^{i}=\frac{x_{i}\,x_{i}^{⊤}}{t\,r\,a\,c\,e\,\left(x_{i}\,x_{i}^{⊤}\right)}$$

in which ⊤ presents the transpose of matrix. The objective function *J* of CSP to optimize the spatial filter follow Eq. 2 and *w* ∈ *R*^*N*×*N*^ is spatial filter:


(2) $$J_{C\,S\,P}\,\left(w\right)=\frac{w^{⊤}\,C^{1}\,w}{w^{⊤}\,\left(C^{1}+C^{2}\right)\,w}$$

We used filters to generate features in two ways depending on the purpose. The first feature *f*_*i,j*_ follows Eq. 3 given as a feature calculation method to calculate accuracy.


(3) $$f_{i,j}=\log\left(w_{j}^{⊤}\,x_{i}\,x_{i}^{⊤}\,w_{j}\right)$$

in which *j* = {1,…,*m*} present the dimensional feature vector, and the first and last *m* rows of the CSP filter maximize the features of each class by the objective function. The second feature ${\bar{f}}_{i,j}$ is the normalized feature, which is divided by the number of samples *S* to consider the sampling rate difference between the datasets, and follows Eq. 4:


(4) $${\bar{f}}_{i,j}=\frac{w_{j}^{⊤}\,x_{i}\,x_{i}^{⊤}\,w_{j}}{S}$$

We compared the eight features vector generated from the CSP filter to analyze the dataset’s compatibility. The upper filter maximizes the left-hand imagery, while the lower filter maximizes the right-hand imagery. We selected the two upper and two lower filters, and the left- or right-hand signals were projected on the filters to yield eight features. We extracted the features and calculated the accuracies for each subject, and the same subject was considered a different subject if measured on any other day. Therefore, the number of subjects in some data increased compared to the results Kaya et al. (2018), Lee et al. (2019), and Stieger et al. (2021) reported previously.

Because each dataset has a different reference electrode, the raw signal was re-referenced through a common average reference, and then bandpass filtered by 8–35 Hz. This frequency band was chosen to cover a sufficient interval (Alpha and Beta rhythms) and was based on the literature (Naeem et al., 2009; Bai et al., 2014; Cho et al., 2017). The segmented and released datasets, they were filtered for each trial, and the data released with continuous raw signals were filtered and then subjected to epoching. The signal was segmented from 500 to 3000 ms based on the cue (onset). The window size may vary in the optimal period for each dataset, but this was chosen for common conditions because the maximum window size obtained from Ahn et al. (2013a) was up to 3 s. Cho et al. (2017) had an exceptionally additional pre-process, and the signal was divided by the signal gain value of 32.

We also selected four filters to extract features to calculate accuracy, divided each dataset into ten sets, and used seven to train and three to test a classifier model. Here, the CSP filter was constructed using only training data. We reported mean accuracy by shuffling the data every time, repeating this process ten times, and the classifier model used linear discriminant analysis (LDA) (Mika et al., 1999). We used t-distributed stochastic neighbor embedding (t-SNE), a non-linear dimension reduction technique that show into low-dimensional space to visualize high-dimensional CSP features (van der Maaten and Hinton, 2008). As it maps data to minimize the difference between the probability distributions in a high and low-dimensional space, the similarity relation between points is maintained while the dimensionality is reduced simultaneously.

## 3. Results

### 3.1. Dataset specifications

This review divided the specifications into public, environmental, and experimental specifications. We defined them by naming them intuitively, as several papers often use different words to describe the same specification. Each definition is in the paper, and a complete list of definitions is provided in the Supplementary material.

#### 3.1.1. Public and environmental specifications

Table 1 shows the public and environmental specifications of the MI and ME dataset, based on the reference in which they were published. The platforms that included the dataset targeted most in this study were 12 in *MOABB*, six in *BNCI Horizon*, six in *Scientific data*, six in *Deep BCI*, three in *Gigascience*, and two in *IEEE DataPort*. A popular EEG device was *BrainProduct* (*N* = 9), followed by *g.tec* (*N* = 5), *Neuroscan* (*N* = 5), *Biosemi* (*N* = 2), and others (*N* = 7). The mean number of electrodes was 49.71, the mean sampling rate was 632.14 Hz, and the electrodes were set based on the international 10−20 (*N* = 13), 10−10 (*N* = 3), or 10−5 (*N* = 2) system. In most cases, the file format of “mat” (*N* = 17) available on MATLAB was released, and in addition, “gdf,” “dat,” “set,” “edf,” “cnt,” or “vhdr” were also released in the format available in the MATLAB EEGLAB toolbox. Zhou (2020) used the “npz” data format that was available in Python.

**TABLE 1:** Public and environmental specifications of motor imagery/execution datasets (–: no information provided).

		Public specifications		Environmental specifications						Essential specifications			
	**References**	**Resources**	**Num. of citations**	** Device**	**Num. of electrodes**	**Extra** **electrode**	**Electrode setting**	**Sampling rate (Hz)**	**Data** **format**	**Signal continuity**	**Event type**	**Event latency**	**Channels**
Motor imagery	Stieger et al., 2021	Scientific data	11	Neuroscan SynAmps	64	Cursor	10−10	1,000	mat	x	o	o	o
Motor imagery, Motor execution	Jeong et al., 2020 *	Deep BCI, Gigascience	25	BrainProduct BrainAmp	60	EOG, EMG	−	2,500	mat	o	o	o	o
Motor imagery	Zhou, 2020	IEEE DataPort	−	Neuroscan SynAmps2	26, 41	EOG	−	500	npz	o	o	o	o
	Wu, 2020	IEEE DataPort	−	Neuroscan SynAmps2	122	ear-EEG	10−20	1,000	dat	o	o	o	o
	Ma et al., 2020	Scientific data	11	Neuroscan SynAmps2	64	EOG, EMG	−	1,000	mat, cnt	o	o	o	o
	Lee et al., 2019	Deep BCI, Gigascience, MOABB	171	BrainProduct BrainAmp	62	EMG	10−20	1,000	mat	o	o	o	o
	Kim et al., 2018	Deep BCI	113	BrainProduct BrainAmp	30	−	−	250	vhdr	o	o	o	o
Motor imagery, Motor execution	Kaya et al., 2018 *	Scientific data	84	Neurofax EEG-1200	19	−	10−20	200	mat	o	o	o	o
	Ofner et al., 2017 *	BNCI Horizon, MOABB	147	g.tec USBamp	61	EOG, EMG	−	512	gdf	o	o	o	o
Motor imagery	Cho et al., 2017	Deep BCI, MOABB, Gigascience	172	Biosemi	64	EMG	10−20	512	mat	x	o	o	o
	Lee et al., 2016	Deep BCI	14	BrainProduct BrainAmp	70	EOG, EMG	10−20	1,000	vhdr	o	x	o	o
	Shin et al., 2017	MOABB	135	BrainProduct BrainAmp	30	NIRS, EOG, ECG	10−5	1,000	mat	o	o	o	o
	Zhou et al., 2016	MOABB	32	−	14	−	10−20	250	cnt	o	x	o	o
	Steyrl et al., 2016	BNCI Horizon, MOABB	93	g.tec USBamp	15	−	10−10	512	mat	o	o	o	x
	Yi et al., 2014	MOABB	46	Neuroscan SynAmps2	64	−	10−20	1,000	mat	x	o	x	x
	Ahn et al., 2013a	Deep BCI	82	Biosemi, BrainProduct BrainAmp	19	−	10−10	512, 500	mat	Δ	Δ	Δ	Δ
	Faller et al., 2012	BNCI Horizon, MOABB	138	g.tec USBamp	13	−	10−5	512	mat	o	o	o	x
	Tangermann et al., 2012	BNCI Horizon, MOABB	652	−	22	EOG	10−20	250	gdf	o	o	o	o
	Grosse-Wentrup et al., 2009	MOABB	178	BrainProduct BrainAmp	128	−	10−20	500	set	o	o	o	o
	Leeb et al., 2007	BNCI Horizon, MOABB	486	g.tec USBama	3 (Central)	EOG	−	250	mat	o	o	o	o
Motor imagery, Motor execution	Schalk et al., 2004 *	MOABB	2,915	−	64	−	10−20	160	edf	o	o	o	o
Motor execution	Schwarz et al., 2020	BNCI Horizon	19	g.tec USBamp	58	EOG, Force-sensing resistor sensor	−	256	mat	o	o	o	o
	Schwarz et al., 2020	BNCI Horizon	19	EEG-VersatileTM system	32	EOG, photodiode sensor	−	256	mat	o	o	o	o
	Schwarz et al., 2020	BNCI Horizon	19	EEG-HeroTM headset	11	photodiode sensor	10−20	256	mat	o	o	o	o
	Wagner et al., 2019	Scientific data	8	g.tec USBamp	108	EMG, EOG, goniometers	10−20	512	set	o	o	o	o
	Brantley et al., 2018	Scientific data	20	BrainProduct BrainAmp	60	EOG, EMG	10−20	1,000	mat	o	−	−	o
	Luciw et al., 2014	Scientific data	87	BrainProduct BrainAmp	64	EMG	−	500	mat	o	−	−	o

For essential specifications, o, satisfied, △, partially satisfied, and x, unsatisfied. *These datasets contain both motor imagery and an execution paradigm.

Many studies have adopted electrooculography (EOG) electrodes (*N* = 12) that can be used to process EEG noise and electromyography (EMG) electrodes (*N* = 9) to track motion. In particular, in addition to EMG, the ME paradigm dataset necessarily has sensors that can quantify movements, such as a photodiode sensor and a force-sensing resistor sensor. When simply comparing the mean number of citations in the same period (2004’∼2020’), the mean of MI datasets (*N* = 86.92) was higher than that of ME datasets (*N* = 33.5).

The essential specifications refer to the minimum component that the public dataset defined in this paper should have, and include signal continuity, events (type and latency), and channels. The signal continuity indicates whether the signal is released as a continuous signal rather than as a segmented signal according to the trial, and the event type presents a class name (e.g., left or right) rather than numbers alone. Triangles indicate cases in which some data do not satisfy these conditions.

#### 3.1.2. Experimental specifications

Table 2 shows the MI dataset’s experimental specifications, based on the published reference. The paradigms of these datasets consisted of the left- and right-hand (*N* = 16), feet or foot (*N* = 9), both hands (*N* = 3), right hand without left hand (*N* = 3), tongue (*N* = 2), and others (*N* = 3). As the type of cue was simple, conveying information was also simple. The cue (onset) is presented to subjects by simple arrows (*N* = 14), text (*N* = 3), objects (moving ball and bar, *N* = 3), or symbolic pictures (hand and foot figure, *N* = 2). Some datasets include online experiments that provide feedback using data obtained from previous sessions (*N* = 6).

**TABLE 2:** Experiment specifications of motor imagery datasets (–: no information provided).

References	Class type	Instruction method	Feedback (online)	Cue type	Average age	Number of						
						Subjects	Males	Classes	Trials per class	Sessions	Runs per session	Trials per run (regardless of classes)
Stieger et al., 2021	Left/right/both hand	EMI	Y	Object	39	62	26	3	3,150∼4,950	7∼11	6	−
Jeong et al., 2020	Arm-reaching (forward/ backward/ left/ right/ up/ down), hand-grasping (cylindrical/ spherical/ lateral), wrist-twisting (pronation/supination)	EMI	N	Arrow, Figure	28	25	10	11	300	3	−	550
Zhou, 2020	Left/right hand, feet, idle	SMI	N	Arrow	23.2*	20	11	4	420	7	6	40
Wu, 2020	Left/right hand	EMI	N	Arrow	25	6	2	2	80^†^	−	10	16
Ma et al., 2020	Right hand, right elbow	KMI	N	Text	23	25	19	2	100	1	5	40
Lee et al., 2019	Left/right hand	EMI	Y	Arrow	29.5	54	29	2	100	2	2	50
Kim et al., 2018	Left/right hand, right foot	SMI	N	Arrow	28*	12	11	3	30	−	−	−
Kaya et al., 2018	Left/right hand, left/right foot, tongue, five fingers	EMI	N	Figure	27.5	13	8	10	−	−	3	300
Ofner et al., 2017	Elbow flexion/extension, forearm supination/pronation, hand open/close	KMI	N	−	27*	15	6	6	120	2	10	42
Cho et al., 2017	Left/right hand	EMI	N	Text	24.8*	52	33	2	100^†^	1	5	40
Lee et al., 2016	Left/right hand, foot	SMI	N	Arrow	26*	52	28	3	50	1	−	−
Shin et al., 2017	Left/right hand	KMI	N	Arrow	28.5*	29	15	2	30	1	3	20
Zhou et al., 2016	Left/right hand, foot	SMI	N	Arrow	25	4	1	3	150	3	2	75
Steyrl et al., 2016	Right hand, feet	KMI	Y	Arrow	25	13	−	2	80	1	8	20
Yi et al., 2014	Left/right/both hands, feet, left hand with right foot, right hand with left foot	KMI	N	Text	24	10	3	6	90	1	9	60
Ahn et al., 2013a	Left/right hand	SMI	N	Arrow	25.3	10	8	2	60	1	3	40
Faller et al., 2012	Right hand, feet	KMI	Y	Arrow	24.8*	12	7	2	100	5	−	40
Tangermann et al., 2012	Left/right hand, feet, tongue	SMI	N	Arrow	−	9	−	4	144	2	6	48
Grosse-Wentrup et al., 2009	Left/right hand	EMI	N	Arrow	25.6*	10	8	2	150	−	−	−
Leeb et al., 2007	Left/right hand	KMI	Y	Arrow (offline)	24.7*	9	6	2	240	2	6	40
				Object (online)					240	3	4	40
Schalk et al., 2004	Left/right/both hands, feet	EMI	Y	Object	−	109	−	4	−	−	−	−

For instruction method, KMI, kinetic movement instruction; EMI, explicit movement instruction; SMI, simple movement instruction.

*The average was manually calculated since only the range of ages are provided in the study.

^†^The number of trials per class for the whole session reported were based on more than half of the subjects.

The number of subjects ranged from 4 to 109, with an mean of 26.23 ± 25.93 for all datasets and an mean of 22.10 ± 18.15 excluding Schalk et al. (2004). In that reference, if the subjects’ age was given only as the range, the mean of the ranges was reported. The subjects’ ages ranged from 23 to 39 years, and the mean age was 26.52 ± 3.52 years. The experiments were conducted largely with young adult males and females. The mean proportion of male subjects was 55.68%, which was balanced according to the average of all datasets, but the proportion of men and women within the dataset was often not balanced.

Because the act of imagining can be ambiguous, experimenters have tried to clarify imagination methods often to help the subjects. We divided the instruction method into three types. If the reference included the instruction to imagine muscle movements or to engage in kinetic motor imagery clearly, it was classified as kinetic movement instruction (KMI, *N* = 7). It was classified as explicit movement instruction (EMI, *N* = 8) if the imaginary behavior was described accurately in detail, such as opening and closing the hands. On the other hand, if only the hand direction was mentioned, it was classified as simple movement instruction (SMI, *N* = 6).

Each paper defined the experimental block with various names, but we used the term adopted commonly in most papers. The session is an experiment a subject performs on different days with the same paradigm, and the “run” is a block performed for a day, although it has a rest interval of several minutes, considering the subject’s condition. The number of trials per class for the entire session reported in Table 2 was based on more than half of the subjects. For example, Wu (2020) reported 80 data points because approximately 83% (*N* = 5) of subjects had 80 trials, and approximately 17% (*N* = 1) had 40 trials. However, Stieger et al. (2021) described it as a range because the number of trials was composed evenly, and the mean value of the range was used to calculate the mean number of trials in all datasets. We attempted to avoid confusion by marking information that was not presented clearly in the source of each dataset as unknown (−).

The number of trials per class for the entire session on a single subject ranged from 30 to 4,950, with mean of 331.2 ± 880.71. In the experiment, 11 datasets were divided into several sessions, seven of which were conducted for 1 day (session), and five were unknown. The mean number of runs per session was six, and the mean number of trials per run was 57 ± 66.42, including all classes. The product of the number of runs per session and trials per run represents the total number of trials conducted per day. For example, Wu (2020) performed ten runs of 16 trials and thus obtained 160 trials per day. This is the sum of all classes, and as its dataset consists of one session, the number of trials per class for the entire session was 80.

#### 3.1.3. Specification analysis

**FIGURE 1:**
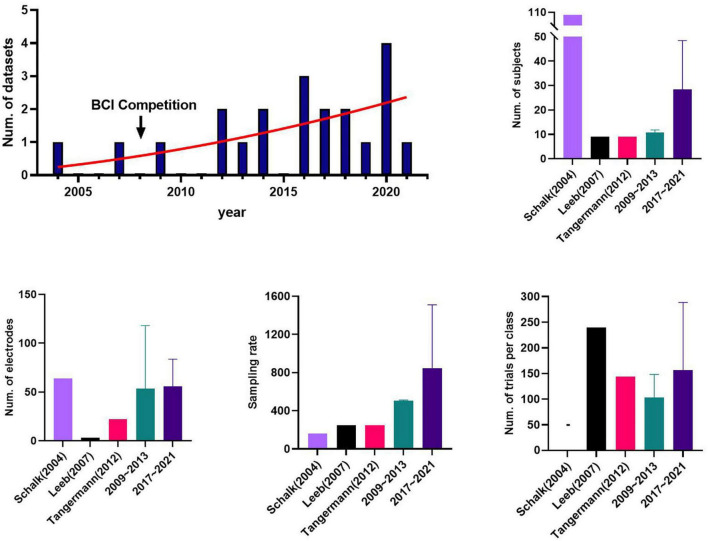
The information of public datasets by years: brain-computer interface (BCI) competition datasets, Leeb et al. (2007) and Tangermann et al. (2012), the old datasets (2009∼2013); and the recent datasets (2017∼2021). Schalk et al. (2004), called PhysioNet project, was excluded due to its exceptionally large number of subjects and being too outdated. Stieger et al. (2021) was excluded from the recent datasets group only in calculating the number of trials per class. The error bar represents the standard deviation.

Figure 1 shows the progression of publishing public datasets for motor imagery with respect to the number of publications and components based on the datasets collected. The number of public datasets published has increased since 2004, particularly in the last 5 years. In addition, we divided the MI data collected into groups to analyze trends over time: the BCI competition datasets; the old datasets (2009–2013), and the released most recently datasets (2017–2021) based on 5 years. The BCI competition datasets, Leeb et al. (2007) and Tangermann et al. (2012), were analyzed separately because they are the datasets the most widely used in many studies, and the old datasets did not include them, although the periods overlap.

We investigated the way the components of the datasets have changed over time because they can vary depending on how the BCI fields grow and the research focus. The MI dataset released recently had a relatively large number of subjects, with an average of 28.40, followed by 10.67 for the old datasets and nine for the BCI competitive dataset. The mean number of electrodes and sampling rate were 57.2 and 847.4 Hz, 45.5 and 443.5 Hz, and 3 and 250 Hz for the recent, old, and BCI competition datasets, respectively. For the mean number of trials per class for the entire session, BCI competition, recent, and old datasets contained 192, 187.5, and 103.3 trials, respectively. We note that the mean number of trials for the recent datasets is 616.67 when Stieger et al. (2021) is included, but it was excluded here to avoid bias.

**FIGURE 2:**
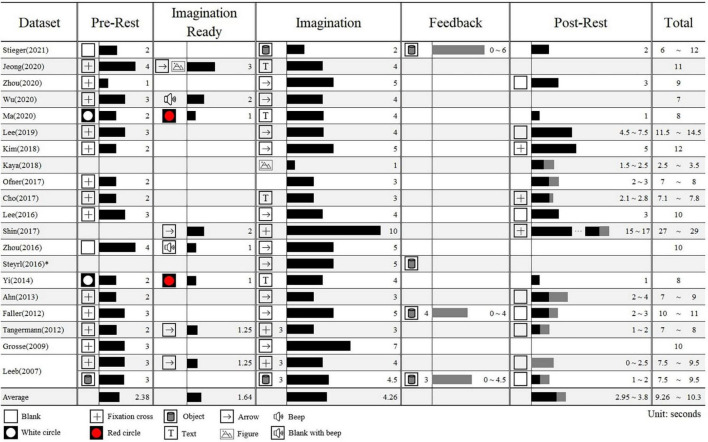
The block information of a trial in each motor imagery dataset. The timings are based on the bagging of pre-rest (0 s). The number next to the stimuli represents the timing when it appeared and the number next to the bar represents the duration of the block in seconds. The gray bar indicates the adjustable time within sub-blocks. *Only the duration of the imagination is provided due to the lack of information in Steyrl et al. (2016).

Figure 2 shows the block’s duration and stimuli type that constitute each dataset’s paradigm based on the reference. MI paradigms are distinguished by five blocks: pre-rest, imagination ready, imagination, feedback, and post-rest. All datasets took the form of pre-rest, imagination, and post-rest, but there was a difference in the latency. We reported only visual stimuli and omitted sound stimuli except those used to indicate differences between sub-blocks.

The length of one trial differed considerably from at least 2.5−29 s, with a mean of 9.8 s. The pre-rest, a sub-block in which subjects have help to gaze their eyes or take a break before the imagination, was 2.38 s on average, and the fixation cross was presented most often (*N* = 13). Some data have sub-block to call attention immediately before the imagination, which prepares for imagination by instructing the class or presenting a stimulus different from the pre-rest. This had mean of 1.64 s. The imagination block ranged from 1 to 10 s, and averaging 4.26 s. There were some datasets in which feedback continued from the imagination block until the brain wave reached specific thresholds. The post-rest was 3.38 s on average, and unlike pre-rest, blank stimuli were often presented. In some cases, ITIs were set differently and, the intervals between stimuli were randomized to prevent subjects from predicting when the next stimulus would be presented.

Table 3 shows the experimental specifications in ME datasets based on data the reference published. For datasets that performed both MI and ME, such as Schalk et al. (2004), Ofner et al. (2017), and Jeong et al. (2020), both were performed with the same paradigm for comparison. Because it is challenging to design a self-paced MI paradigm without an onset cue, Kaya et al. (2018) performed a self-paced ME in which only two subjects pressed the button. In the case of ME, the paradigm consisted of largely more complex and dynamic motion than MI. For the subjects to perform the motion task, most of the cue displays were presented with actual objects (*N* = 4).

**TABLE 3:** Paradigm specifications of motor execution datasets (–: no information provided).

References	Class type	Cue type	Average age	Number of		
				Subjects	Males	Classes
Schwarz et al., 2020	Palmar grasp, lateral grasp	Object	22.5*	45	24	2
Jeong et al., 2020	Hand (forward, backward, left, right, up, down), hand-grasping (cylindrical, spherical, lateral), wrist-twisting (pronation, supination)	Figure	28*	25	10	11
Wagner et al., 2019	Walking, preferred cadence walking, acceleration/deceleration walking	Auditory	29.1	18	11	4
Brantley et al., 2018	Level ground walking, stair descent, stair ascent, ramp descent, ramp ascent	Object	24.5*	10	5	5
Kaya et al., 2018	Left/right hand (bottom press)	No (self-paced)	27.5*	2	2	2
Ofner et al., 2017	Elbow flexion/extension, forearm supination/pronation, hand open/close	−	27	15	6	6
Luciw et al., 2014	Prevailing contact surface (sandpaper, suede, silk) × weight (165 g, 330 g, 660 g)	Object	27*	12	5	9
Schalk et al., 2004	Open and close left/right fist, Open and close both fists, feet	Object	−	109	−	4

*The average was manually calculated since only the range of ages are provided in the study.

The number of subjects varied from 2 to 109, with a mean overall of 29.5 ± 34.57 and a mean of 18.14 ± 13.80 excluding Schalk et al. (2004). The mean the subjects’ ages in each dataset ranged from 22.5 to 29.1 years, and the average age overall was 27.18 ± 1.53 years. The mean proportion of male subjects was 55.15%, but the proportion of males and females in a single dataset was not balanced. Many experiments were conducted continuously on the ME dataset, and it was often difficult to distinguish trials. Therefore, we did not track paradigm information in detail, such as the MI datasets in Table 2.

### 3.2. Assessment of classification accuracy and compatibility between other datasets

Table 4 presents the brief recording settings of representative datasets for CSP analysis based on actual data that can be mismatched from what the references reported. The table consists of the parameters that may affect the compatibility between the datasets, such as device, paradigm, and the parameters. The signal power indicates the mean of the signal envelope of the pre-processed Cz channels during the entire session. However, in Ahn et al. (2013a), seven measured by *Biosemi* were calculated as the mean of the entire channel because there was no channel information. It can infer the range of amplitudes in each dataset.

**TABLE 4:** The brief recoding settings of representative datasets.

References	Num. of sessions	Device (num. of EEG electrodes)	Reference electrode	Ground electrode	Num. of trials		Instruction method	Cue type	Signal power^†^
					L	R			
Stieger et al., 2021	589*	Neuroscan SynAmps (60)	Ref	−	110∼114	110∼115	EMI	Object	4.70 ± 17.59
Lee et al., 2019	108*	BrainProduct BrainAmp (62)	Nose	Afz	200	200	EMI	Arrow	3.28 ± 1.52
Kim et al., 2018	11	BrainProduct BrainAmp (32)	FCz	Fpz	30	30	SMI	Arrow	2.89 ± 0.95
Kaya et al., 2018	43*	Neurofax EEG-1200 (19)	−	A1, A2	138∼477	144∼483	EMI	Figure	2.71 ± 0.75
Cho et al., 2017	52	Biosemi (64)	−	−	100∼120	100∼120	EMI	Text	3.61 ± 0.94
Shin et al., 2017	29	BrainProduct BrainAmp (30)	Mastoids	Fz	60	60	KMI	Arrow	3.21 ± 1.04
Yi et al., 2014	10	Neuroscan SynAmps2 (64)	Nose	Pre-frontal lobe	70∼80	70∼80	KMI	Text	7.75 ± 5.30
Ahn et al., 2013a	10	Biosemi (19), BrainProduct BrainAmp (19)	−	−	60	60	SMI	Arrow	4.31 ± 1.07

*Since a single subject had several sessions in this dataset, there is a difference in the number of subjects in Table 1.

^†^The signal power presents the mean of the pre-processed signal envelope during the entire session.

**FIGURE 3:**
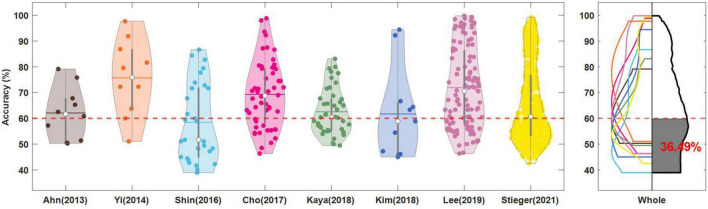
Classification accuracies of left-right motor imagery. In each dataset, the filled dot represents the individual subject’s accuracy and the average (horizontal line) and median (white circle) of the accuracies are presented. The dotted red line denotes the threshold accuracy (60%) for identifying the low performers (BCI illiterates). In the right panel, the accuracy distributions obtained by Kernel Density Estimation are overlaid and the average of the normalized accuracy distributions is presented in the black lines. The area of low performers based on the threshold accuracy is highlighted in black color and the estimated percentage of the population is written. Kim et al. (2018) was excluded from obtaining the average of normalized accuracy distribution.

As shown in Figure 3 and Yi et al. (2014) had the highest accuracy of 75.71 ± 14.69%, followed by 71.92 ± 15.70% for Lee et al. (2019), 69.27 ± 13.03% for Cho et al. (2017), 65.65 ± 15.17% for Stieger et al. (2021), 62.54 ± 9.01% for Kaya et al. (2018), 62.08 ± 10.20% for Ahn et al. (2013a), 62.47 ± 17.22% for Kim et al. (2018) and 58.45 ± 15.17% for Shin et al. (2017). Yi et al. (2014), Cho et al. (2017), and Lee et al. (2019) have high accuracy and the distribution of accuracy forms a normal distribution, including both good and poor performers.

The MI-BCI poor performer is typically one who demonstrates a 60%–70% accuracy (Ahn et al., 2013b), and we considered the minimum critical criterion 60%. Yi et al. (2014) had the lowest rate of 20%, followed by 28.9% for Cho et al. (2017), 29.63% for Lee et al. (2019), 40.0% for Ahn et al. (2013a), 46.51% for Kaya et al. (2018), 49% for Stieger et al. (2021), 54.55% for Kim et al. (2018), and 62.1% for Shin et al. (2017). The grand mean accuracy of 861 subjects (or sessions) was 66.28%, and the BCI poor performer ratio was 45.30%. However, of 861 subjects, Stieger’s work included 589, which may have biased the grand mean. We calculated the mean of each dataset’s normalized accuracy distribution (right panel of Figure 3) was 66.49%, and the BCI poor performer ratio, the black-filled area, was 36.49%.

**FIGURE 4:**
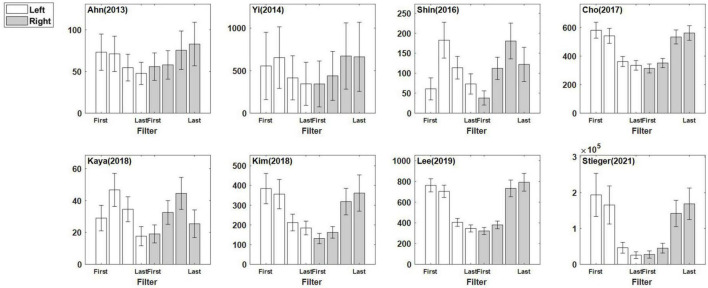
Eight common spatial pattern features for each dataset. The four CSP filters were applied to left trials and right trials, and the eight features were concatenated. The presented features are the averaged features over all sessions/subjects in each dataset. Note that the first two CSP filters are designed to maximize the variance of left motor imagery trials, and the last two CSP filters does that for the right motor imagery trials. The error bar represents the standard error.

Figure 4 illustrates eight CSP features for each dataset that are the grand mean of all subjects within the dataset overall. This figure indicates directly how well the CSP filter constructed the features to maximize the characteristics of each class in each dataset. Although the pattern of the features was similar according to the filter, the feature scales differed for each dataset.

**FIGURE 5:**
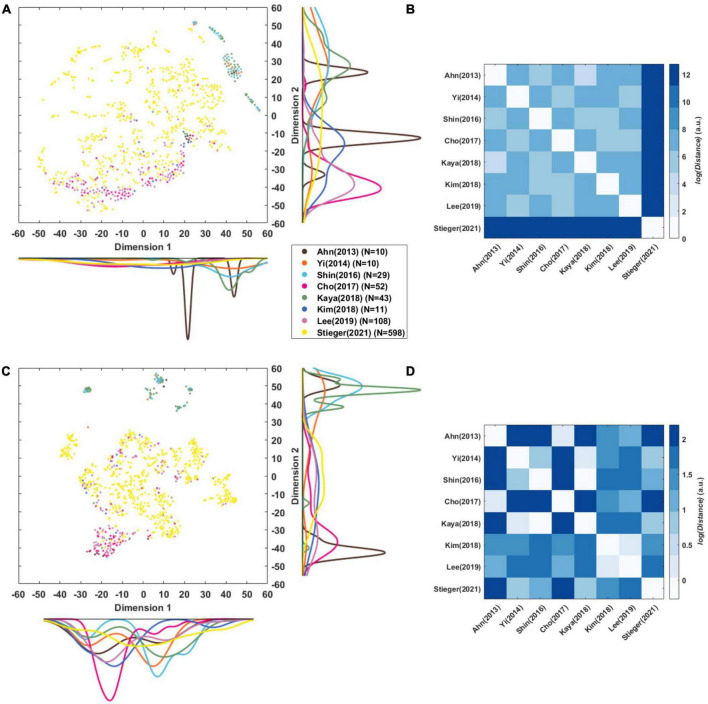
Visualization of CSP features. **(A,C)** t-SNE maps of eight-dimensional raw CSP features and normalized CSP features of each subject that was compressed into a two-dimensional space. The color lines along each axis display the marginal distribution of each dimension computed by Kernel Density Estimation. **(B,D)** Euclidean distance (in arbitrary units) among centroids of datasets in the eight-dimensional raw CSP features’ and normalized CSP feature’ space.

Figure 5A presents a t-SNE map of the eight-dimensional CSP features using the Mahalanobis distance. The reason why it was used here is that we examined the distribution between the datasets considering the distance of each subject. Each circle is a single subject or session, and the marginal distribution represents the distribution of datasets and the relation between them datasets. As there is a scale difference between the features (Figure 4), it was remapped to normalize them by variance (Figure 5C). This mitigates the difference in the scale between datasets, and it is possible to confirm the t-SNE map based on the features’ pattern.

Stieger et al. (2021) was widely distributed in the t-SNE maps of CSP features, while the other datasets were generally cohesive between same datasets (Figures 5A, C). There were cases in which the distributions of datasets overlapped, such as Cho et al. (2017) with Lee et al. (2019), and Shin et al. (2017) with Kaya et al. (2018) and Yi et al. (2014). Ahn et al. (2013a) forms two clusters, and Kim et al. (2018) is distributed widely compared to the number of data points.

Figures 5B, D represents the Euclidean distance between datasets based on the center points as each dataset’s mean in the eight-dimensional features’ space. This was used here rather than the Mahalanobis distance because the straight-line distance of each dataset’s central point was obtained. It is the distance in the feature space, so the unit and the size of the number are meaningless and can be interpreted as a relative size.

In the distance between the raw CSP features (Figure 5B), the mean of the log distance value from the other datasets was 12.75 for Stieger et al. (2021), 7.56 for Lee et al. (2019), 7.41 for Yi et al. (2014), 7.36 for Kim et al. (2018), 7.35 for Cho et al. (2017), 7.31 for Kaya et al. (2018), 7.26 for Shin et al. (2017), and 7.21 for Ahn et al. (2013a). The dataset closest to each other were as follows: 4.61 between Ahn et al. (2013a) and Kaya et al. (2018), 5.20 between Shin et al. (2017) and Ahn et al. (2013a), 5.45 between Yi et al. (2014) and Cho et al. (2017), 5.58 between Lee et al. (2019) and Yi et al. (2014), 6.2 between Kim et al. (2018) and Shin et al. (2017), and 12.75 between Stieger et al. (2021) and Lee et al. (2019).

In the distance between the normalized CSP features (Figure 5D), the mean of log distance value from the other datasets was 1.57 for Ahn et al. (2013a), 1.56 for Cho et al. (2017), 1.34 for Stieger et al. (2021), 1.332 for Shin et al. (2017), 1.31 for Lee et al. (2019), 1.29 for Kim et al. (2018), 1.27 for Yi et al. (2014), and 1.21 for Kaya et al. (2018). The datasets closet to each other were as follows: −0.272 between Shin et al. (2017) and Kaya et al. (2018), 0.19 between Ahn et al. (2013a) and Cho et al. (2017), 0.24 between Kim et al. (2018) and Lee et al. (2019), 0.29 between Yi et al. (2014) and Kaya et al. (2018), 0.67 between Stieger et al. (2021) and Yi et al. (2014).

## 4. Discussion

### 4.1. Review dataset specifications

The number of public datasets has been increasing recently, but for them to be used actively, researchers need sufficient information to be available for comparative selection. Therefore, we scrutinized the details of datasets, which have been covered before rarely. This provides guidelines to which researchers can refer when using public datasets and confirms the trends in movement-related studies.

Among the datasets collected, the MI datasets were more numerous than the ME datasets, and both were focused on the MI paradigm (Table 1). Schalk et al. (2004) had the only data with more than 100 subjects for MI and ME. Except for that datum, there were only four MI datasets with 50 or more, and the remainder had 30 or fewer. Schwarz et al. (2020) had 30 or more ME datasets. It was confirmed that datasets that recruited far more subjects than BCI competitive datasets were released.

We compared and analyzed the BCI competitive datasets that are used frequently and the groups of datasets based on the public year they were released publicly (Figure 1). Although the number of samples is insufficient to analyze the trends by year, research trends can be confirmed by comparing BCI competitions released approximately 10 years ago, datasets released at that time, and those released recently.

We found that not only has the number of published datasets increased each year, but also the size of a single dataset has increased with respect to the number of subjects, electrodes, and sampling rate. Recent datasets include a larger mean number of trials than do old ones, and the datasets in 2020 and 2021 consisted often of multi-sessions measured over several days. In particular, the number of subjects per dataset has also increased in the recent datasets. Compared to the number of BCI competitive datasets with 9 subjects, the mean number of subjects in datasets in the past 5 years was 29.5, a significant increase. The dataset released recently tend to collect big data because data-driven deep learning and machine learning are used actively now in BCI research (Altaheri et al., 2021).

In the measurement environment, a relatively heavy device was preferred to a lightweight one. However, recently, an increasing number of BCI research studies are using lightweight EEG devices. LaRocco et al. (2020) reported the possibility of drowsiness detection studies based on lightweight EEGs, such as MindWave, EmotionalEpoc, and OpenBCI, and Krigolson et al. (2017) quantified ERP using a portable MUSE EEG system. On the other hand, high-cost, high-resolution EEG devices are relatively preferred in the MI and ME research studies. The datasets published more recently show a trend toward higher sampling rates and a greater number of electrodes. The public datasets were also measured in whole brain regions, except for Leeb et al. (2007) and Tangermann et al. (2012), which released data from constrained environments for competition purposes.

In addition, MI and ME had differences in the difficulty of conducting experiments by subjects. MI included primarily simple and discrete tasks because it is difficult when confirming the conduction of the work, while ME consists of relatively complex tasks that are performed as delicate movements, such as the object’s weight and tactile differences. Because both paradigms are sensitive to noise due to movement, additional electrode measurements have been preferred rather than lightening the environment in practical terms. Many datasets also measured electrodes capable of post-processing noise, such as EOG and EMG electrodes, and in particular, all ME datasets included motion-tracking sensors. This trend overall requires signals obtained with reliable EEG devices, as biomarker extraction in MI-BCI is complex and requires as many EEG resources as possible for accurate analysis (Lee et al., 2019).

### 4.2. Importance of paradigm parameters

The basic framework of the current MI paradigm is a cue-based format in which a subject gazes at the fixation cross (pre-rest), instructions are presented, such as directions (cue), and then imagination is conducted for a certain period. There are often paradigms in which feedback from images is presented using models learned from data obtained during calibration sessions (Mousavi et al., 2017). Many MI studies follow the conventional Graz BCI motor imagery paradigm (Pfurtscheller et al., 1993). Thus far, information about paradigms in MI experiments has not been considered important. The MI paradigm is fixed only in composition; according to this investigation, there are no specific established details.

Each experimental parameter can influence brain activity as a form of stimulus, and ultimately is likely to lead EEG signal differences between datasets, even when based on the same paradigm. The public datasets collected had enormous differences in the details about the paradigm parameters, such as block duration and cue type (Figure 2 and Table 2). The stimulus presented on the screen during the break before and after imagination largely used a fixation cross or blank. They could be used as a stimulus to minimize the effect and serve merely for eye fixation. There were various cue types in the imagination block: 14 for arrow; 3 for text; 3 for object; and 2 for figure. The length of the imagination block ranged from 1 to 10 s, and the length of one trial varied considerably from a minimum of 2.5 to a maximum of 29 s.

Brain-computer interface (BCI) researchers overlook the method of imagination among parameters often, which can make the differences in brain waves. It has been reported that the EEG of KMI corresponds to sensory imagination performs better in classifying MI than the EEG of visual motor imaging (VMI) corresponds to the visual network (Yang et al., 2021). Among the datasets in this study, seven provided instruction using only class information (SMI), eight clarified movement tasks (EMI), and six instructed the subjects to focus their muscles on movement (KMI). In the case of SMI datasets, information may have been omitted because the matter was not considered an essential factor to researchers.

Practicing the same movement to improve the imagination before the experiment, or helping the subject by clarifying the way to imagine, can also affect the results. Some datasets, such as those of Yi et al. (2014), Cho et al. (2017), and Ofner et al. (2017), explained that the subjects engaged in a practice process before MI, but most datasets cannot confirm this explanation. It was confirmed that the activity in the motor-related area increased more with the method of performing MI after the actual motion, not with a simple verbal instruction method (Hasegawa et al., 2017). Lorey et al. (2009) found that brain activation occurs more strongly in MI from the first-person perspective than the third-person perspective. Thus, researchers can improve MI performance through paradigm details that aid imagination.

We confirmed that the detailed elements of the experimental paradigm differed across datasets, and we believe that these need to be considered because it is quite possible that they affect brain waves. O’Shea and Moran (2017) pointed out that MI and ME research has been conducted continuously according to the motion simulation theory (Jeannerod, 2001), but there is a lack of understanding of the cognitive mechanisms that underlie MI. To study this in more detail, researchers need to share sufficient information about the paradigms they employ. Therefore, in the future, they need to design paradigm details more delicately in MI experiments and describe them in their papers.

### 4.3. Comparing the quality of representative MI datasets

We confirmed the dataset’s quality by comparing the CSP accuracies calculated under the same conditions. There is one limitation, in that we did not calculate each dataset’s optimal accuracy to equalize the conditions. If the length of the CSP input signal is increased further or the frequency band is widened, then some datasets may have increased accuracy. We also agree that the quality of signals and datasets can be quantified in many ways, and it is inappropriate to define quality unconditionally according to only mean accuracy. We evaluated the data’s quality on the assumption that researchers who use public datasets collected high-quality data for MI classifier model generation or explored MI biomarkers, and thus evaluated the data quality based on the mean accuracy and MI-BCI poor performer rate based on a threshold of 60%.

The mean accuracy was good in the order of Yi et al. (2014), Lee et al. (2019), Cho et al. (2017), Stieger et al. (2021), Kaya et al. (2018), Ahn et al. (2013a), Kim et al. (2018), and Shin et al. (2017). The low performer rate was low in the order of Yi et al. (2014), Cho et al. (2017), Lee et al. (2019), Ahn et al. (2013a), Kaya et al. (2018), Stieger et al. (2021), Kim et al. (2018), and Shin et al. (2017). Lee et al. (2019) and Cho et al. (2017) can be considered to have obtained high-quality data because they had a vast number of samples that were distributed normally. The mean accuracy and poor performer rates of the entire datasets were 66.28 and 45.30%, respectively, but this may be a biased result because Stieger et al. (2021) accounted for more than the majority of the entire sample. Therefore, the mean accuracy and poor performer rate obtained from the dataset’s normalized distribution were 66.49% and 36.49%, respectively. A meta-analysis of 861 people in different environments confirmed that approximately one-third of all MI-BCI users were the poor performers.

Poor BCI performer is a challenge that must be solved for BCI technology to develop. As with Kim et al. (2018)’s research objectives, many studies have attempted to improve BCI performance (Vidaurre and Blankertz, 2010), and we found that one-third of the many datasets we collected corresponded to theirs. In addition, although there was a difference in the ratio for each dataset, the fact that all of them included a certain ratio of poor performers suggests that they cannot continue to be excluded simply from the analysis.

When public datasets are used in MI studies, it may be necessary to take poor performers into account and analyze them. It has been found that it is better to use only subjects who have achieved a certain level of performance or more than all subjects to create a generalized model for MI (Won et al., 2021). As in this study, researchers would need to choose the optimal data because they vary depending on the study’s purpose. They can select datasets with a large number of high performers to form an MI-optimized model or datasets with a high proportion of poor performers to analyze their neurophysiological characteristics. In particular, the poor performers will be very important research subjects in future efforts to commercialize BCI. If this is not addressed, one-third of those who attempt to BCI technology will not be able to do so, even if they purchase it, which could be fatal to the interface technology. Because our study evaluated data quality based on accuracy, undervalued datasets may be a suitable sample for this study.

### 4.4. Inferring the compatibility between representative MI datasets

We inferred compatibility between datasets by referring to CSP features extracted under the same conditions. The expectation might be that the closer the distance to the other datasets within the feature space, the higher their compatibility, while the more heterogeneous the distribution, the lower the compatibility. This assumes that the signals can be compared in the same dimension because they were moved to the feature domain using the same methodology, but still reflected dataset-specific characteristics.

Figure 4 shows the difference in the scale of the CSP feature in each dataset that is related to the power value of the pre-processed signal in Table 4. The sum of the mean and the standard deviation of the signal power is more prominent in Stieger et al. (2021) than others, and the scale of the CSP feature is also the largest. Yi et al. (2014) and Lee et al. (2019) showed a relatively similar mean of CSP features value, but there were significant difference in the variance of the CSP features and the signal powers: 3.28 ± 1.52 for Lee et al. (2019) and 7.75 ± 5.30 for Yi et al. (2014). It is expected that the CSP features reflect the signal’s power and the dispersion of the individual data that constitute the dataset. However, the CSP feature is an indicator that is not affected by power alone. Kaya et al. (2018) and Kim et al. (2018) had similar signal power ranges, but there was a large difference in the feature scale. This is because the signal reflects multiple data information in addition to signal power as it transposes to the CSP feature domain.

We analyzed the dataset’s feature distribution using raw features, including each signal’s the power information (Figure 5A). In addition, we examined the distribution based on the pattern using the normalized CSP features (Figure 5C). Individual sessions within the same dataset may have formed clusters because they may be superior to that in other datasets. Although the shape of individual sessions’ (subjects) the distribution in Figures 5A, C differ the overlapping dataset distributions are nearly the same. There is overlap between Yi et al. (2014), Shin et al. (2017), and Kaya et al. (2018), and between Cho et al. (2017), Lee et al. (2019). Interestingly, Ahn et al. (2013a)’s data formed two distribution clusters, which may be due to measurements with two devices. In Figure 5, one cluster overlaps Cho et al. (2017) measured by *Biosemi*, and the other overlaps Shin et al. (2017) measured by *BrainProduct*. This is consistent with the finding that EEG devices can affect the data significantly (Melnik et al., 2017).

This is confirmed more clearly in Figure 5D, which shows the distance between datasets in a more eight-dimensional normalized feature space. Ahn et al. (2013a) and Cho et al. (2017) measured by *Biosemi*, Kim et al. (2018) and Lee et al. (2019) measured by *Brainproducts*, and Yi et al. (2014) and Stieger et al. (2021) measured by *Neuroscan* were the closest datasets to each other. Figure 5B shows that the scale of the signal affects the compatibility between the data greatly. Stieger et al. (2021) has a large signal power compared to other datasets and was the farthest from all other data. It is expected that if the signal scale is solved, the compatibility between datasets measured with the same company’s equipment will be the best.

As explained in section “4.2. Importance of paradigm parameters,” the paradigm parameters, such as the cue type, may affect the distance. Figure 5B shows that Shin et al. (2017) was closest to Ahn et al. (2013a) and Kim et al. (2018) was closest to Shin et al. (2017), who used an arrow stimulus. Yi et al. (2014) was closest to Cho et al. (2017), who a used text stimulus. Figure 5D shows that Kim et al. (2018) was closest to Lee et al. (2019) who used a text stimulus. Another factor is the method of instruction used. Figure 5B shows that Stieger et al. (2021) is closest to Lee et al. (2019), and in Figure 5D and citeBR80 is closest to Kaya et al. (2018), who used the same instruction method as EMI.

Although not confirmed in this study, details of EEG devices, such as electrode types, may affect signal compatibility as well. EEG was sensitive to noise previously, so electrodes were attached with gel to reduce impedance. Recently, however, several pin types have been developed as dry EEG with wet device quality for convenience (Di Flumeri et al., 2019; Heijs et al., 2021). Habibzadeh Tonekabony Shad et al. (2020) confirmed that the input impedance of the latest EEG system of dry electrodes and amplifiers is more than 100 MΩ, while the impedance of conventional wet electrodes is less. The electrode type can affect the signal power because of impedance, and the resulting baseline difference between EEG signals can affect compatibility. However, in our study, we could not confirm this because only some studies provided information about the electrode type, such as Kaya et al. (2018), Jeong et al. (2020), and Ma et al. (2020), who used a gel type electrode.

### 4.5. Recommendations for a public dataset

We found that there are cases in which data are disclosed that lack important details (see Supplementary Material 2). These can have a significant effect on the analysis, and some can lead to distorted results if not taken into account. To avoid this problem, we suggest that BCI researchers give attention to certain considerations when they release future datasets.

The EEG-BIDS project was established to develop a standard format to organize and share brain imaging datasets between laboratories better (Pernet et al., 2019). The format contains sufficient information for data analysis. We believe that BIDS could facilitate better use of public datasets and ultimately foster an open science trend in the field. However, some information may not be available in certain experiments, and collecting all of the information for BIDS or related standard formats may be another burden to researchers. Indeed, among the datasets, only Ma et al. (2020) was provided in BIDS format. Therefore, using a well-organized standard format is obviously encouraged, but what to include is another important issue when it is not an option to use standard. Here, we will discuss the minimal components that researchers should consider when they share their datasets with the public.

Here, we present components that researchers who cannot follow the BIDS form must have when they release data. The list includes only the bare minimum of information to encourage the dataset to be published. The essential components that a dataset must contain are as follows:

• EEG data (continues EEG signal)
The EEG signal shares the continuous raw signal rather than the segmented signal. It takes the form of a matrix of channel x time, and the file format recommends “mat” and “npy.”
• Event information (event type, latency of event)
Event types provide the onset triggers of each block and describe the corresponding information clearly. The event’s latency is the data point at the occurrence of an event consistent with the EEG data.
• Environmental information (electrode, sampling rate)
Electrodes are provided by channel name and location on the scalp for visualization. Sampling rates are provided based on published data and not on the measurement environment.

We recommend sharing both the MATLAB file format, used conventionally in EEG analysis, and the Python file format, whose use is increasing with deep learning research. It is recommended that the signal be released as consecutive signals rather than as separate signals by trial. As there is a concern about the edge effect in the bandpass filtering used commonly for short-length signals in EEG analysis, we encourage opening the raw signal to maximize the possibility of analysis. In addition, this may help infer paradigm specifications, such as ITI, which is not presented to the user through event information. Approximately 71% of the MI and ME datasets collected met this recommendation, and the datasets that can be loaded into the MATLAB, even if they are not “mat” format exceptionally, were included.

**FIGURE 6:**
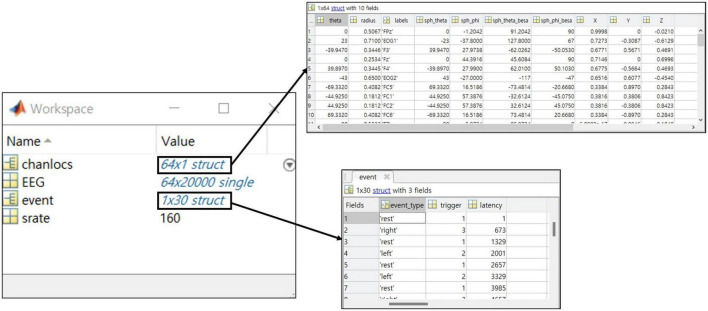
The example of a structure for the recommended dataset with a MATLAB data structure preview.

One crucial point is that all information should be included in the data structure so that experimental information can be confirmed only with a data file alone (Figure 6). Information in collected the public datasets we collected was scattered, for example, papers’ references, separate text or word files, and variables within the data file. To prevent the users from having to make unnecessary efforts, the essential information at least needs to be presented intuitively within the data.

## 5. Conclusion

Brain-computer interface researchers have used public datasets to increase efficiency or to validate their findings. However, until now, they have been using primarily small datasets of classical paradigms that were released 10 years ago. BCI researchers need to move beyond conventional datasets and use the numerous public datasets reported in this study actively. The expansion of data offers objectivity to research, and verification through multiple datasets can increase replicability in various environments. To accomplish these goals, it is necessary to use at least three public datasets and select datasets that consider the environmental factors.

Many labs need to release datasets actively. Other research areas tolerate of open sources through Github already and share data with models, which has created a knowledge explosion. Because BCI is a study of human subjects, it is clear that more caution is needed, but there are advantages that can be achieved through sharing data. In the case of deep learning, pre-training models are already used to improve performance, even in different domains.

Although the paradigm parameters have not been considered as important in conventional MI studies, new consideration is needed because they can influence brain waves and may be related to differences between datasets. Many studies collect subjects’ information, but they are not actively used in relation to brain waves analysis. Even factors beyond the experimenter’s control should be provided with public datasets for researchers to explore.

We believe that this review contributes as a guideline for the use of public datasets on the part of many researchers at this time in BCI research where data are critically important. There have been many review papers on specific research topics in BCI studies, but only a few studies have addressed the data themselves. However, the recent trend in research is changing to data-based research and particularly given that the data themselves are valuable because of the nature of BCI research, this trend could be meaningful and valuable when reviewing the data at this time.

## Author contributions

DG and MA contributed to the conceptualization and drafted and edited manuscript. DG, KW, MS, CN, SJ, and MA contributed to the methodology. DG collected the data. DG, MS, and MA analyzed the data and visualization. All authors read and approved the published version of the manuscript.
