# Corrigendum: Review of public motor imagery and execution datasets in brain-computer interfaces

**DOI:** 10.3389/fnhum.2023.1205419

**Published:** 2023-05-17

**Authors:** Daeun Gwon, Kyungho Won, Minseok Song, Chang S. Nam, Sung Chan Jun, Minkyu Ahn

**Affiliations:** ^1^Department of Computer Science and Electrical Engineering, Handong Global University, Pohang, Republic of Korea; ^2^School of Electrical Engineering and Computer Science, Gwangju Institute of Science and Technology, Gwangju, Republic of Korea; ^3^Edward P. Fitts Department of Industrial and Systems Engineering, North Carolina State University, Raleigh, NC, United States; ^4^Department of Industrial and Management Systems Engineering, Kyung Hee University, Yongin-si, Republic of Korea; ^5^AI Graduate School, Gwangju Institute of Science and Technology, Gwangju, Republic of Korea; ^6^School of Computer Science and Electrical Engineering, Handong Global University, Pohang, Republic of Korea

**Keywords:** brain-computer interface (BCI), motor imagery, motor execution, public dataset, data quality, meta-analysis

In the published article, there were errors in affiliations 1 and 5. Instead of “School of Electrical Engineering and Computer Science, Handong Global University, Pohang-si, Republic of Korea” and “AI Graudate School, Gwangju Institute of Science and Technology, Gwangu, Republic of Korea”, it should be “Department of Computer Science and Electrical Engineering, Handong Global University, Pohang, Republic of Korea” and “AI Graduate School, Gwangju Institute of Science and Technology, Gwangju, Republic of Korea”.

In the original article, there were errors in Table 1. The mark “^*^” was incorrectly used for the dataset “Cho et al., 2017^*^” in the column “References”, the “^*^” should be removed. The Num. of electrodes of the dataset “Zhou, 2020” was “26,41” and the sampling rate of the dataset “Ahn et al., 2013a” was “512,500” which may cause misreading. The texts should be “Cho et al., 2017”, “512, 500” and “26, 41”. The corrected Table 1 appears below.

**Table 1:** Public and environmental specifications of motor imagery/execution datasets (–: no information provided).

		**Public specifications**		**Environmental specifications**						**Essential specifications**			
	**References**	**Resources**	**Num. of citations**	**Device**	**Num. of electrodes**	**Extra electrode**	**Electrode setting**	**Sampling rate (Hz)**	**Data format**	**Signal continuity**	**Event type**	**Event latency**	**Channels**
Motor imagery	Stieger et al., 2021	Scientific data	11	Neuroscan SynAmps	64	Cursor	10-10	1,000	mat	x	o	o	o
Motor imagery, Motor execution	Jeong et al., 2020*	Deep BCI, Gigascience	25	BrainProduct BrainAmp	60	EOG, EMG	–	2,500	mat	o	o	o	o
Motor imagery	Zhou, 2020	IEEE DataPort	–	Neuroscan SynAmps2	26, 41	EOG	–	500	npz	o	o	o	o
	Wu, 2020	IEEE DataPort	–	Neuroscan SynAmps2	122	ear-EEG	10-20	1,000	dat	o	o	o	o
	Ma et al., 2020	Scientific data	11	Neuroscan SynAmps2	64	EOG, EMG	–	1,000	mat, cnt	o	o	o	o
	Lee et al., 2019	Deep BCI, Gigascience, MOABB	171	BrainProduct BrainAmp	62	EMG	10–20	1,000	mat	o	o	o	o
	Kim et al., 2018	Deep BCI	113	BrainProduct BrainAmp	30	–	–	250	vhdr	o	o	o	o
Motor imagery, Motor execution	Kaya et al., 2018*	Scientific data	84	Neurofax EEG-1200	19	–	10–20	200	mat	o	o	o	o
	Ofner et al., 2017*	BNCI Horizon, MOABB	147	g.tec USBamp	61	EOG, EMG	–	512	gdf	o	o	o	o
Motor imagery	Cho et al., 2017	Deep BCI, MOABB, Gigascience	172	Biosemi	64	EMG	10–20	512	mat	x	o	o	o
	Lee et al., 2016	Deep BCI	14	BrainProduct BrainAmp	70	EOG, EMG	10–20	1,000	vhdr	o	x	o	o
	Shin et al., 2017	MOABB	135	BrainProduct BrainAmp	30	NIRS, EOG, ECG	10–5	1,000	mat	o	o	o	o
	Zhou et al., 2016	MOABB	32	–	14	–	10–20	250	cnt	o	x	o	o
	Steyrl et al., 2016	BNCI Horizon, MOABB	93	g.tec USBamp	15	–	10–10	512	mat	o	o	o	x
	Yi et al., 2014	MOABB	46	Neuroscan SynAmps2	64	–	10–20	1,000	mat	x	o	x	x
	Ahn et al., 2013a	Deep BCI	82	Biosemi, BrainProduct BrainAmp	19	–	10–10	512, 500	mat	Δ	Δ	Δ	Δ
	Faller et al., 2012	BNCI Horizon, MOABB	138	g.tec USBamp	13	–	10–5	512	mat	o	o	o	x
	Tangermann et al., 2012	BNCI Horizon, MOABB	652	-	22	EOG	10–20	250	gdf	o	o	o	o
	Grosse-Wentrup et al., 2009	MOABB	178	BrainProduct BrainAmp	128	–	10–20	500	set	o	o	o	o
	Leeb et al., 2007	BNCI Horizon, MOABB	486	g.tec Usama	3 (Central)	EOG	–	250	mat	o	o	o	o
Motor imagery, Motor execution	Schalk et al., 2004*	MOABB	2,915	–	64	–	10–20	160	edf	o	o	o	o
Motor execution	Schwarz et al., 2020	BNCI Horizon	19	g.tec USBamp	58	EOG, Force-sensing resistor sensor	–	256	mat	o	o	o	o
	Schwarz et al., 2020	BNCI Horizon	19	EEG-VersatileTM system	32	EOG, photodiode sensor	–	256	mat	o	o	o	o
	Schwarz et al., 2020	BNCI Horizon	19	EEG-HeroTM headset	11	photodiode sensor	10–20	256	mat	o	o	o	o
	Wagner et al., 2019	Scientific data	8	g.tec USBamp	108	EMG, EOG, goniometers	10–20	512	set	o	o	o	o
	Brantley et al., 2018	Scientific data	20	BrainProduct BrainAmp	60	EOG, EMG	10–20	1,000	mat	o	–	–	o
	Luciw et al., 2014	Scientific data	87	BrainProduct BrainAmp	64	EMG	–	500	mat	o	–	–	o

For essential specifications, o: satisfied, Δ: partially satisfied and x: unsatisfied.

^*^These datasets contain both motor imagery and execution paradigm.

The authors apologize for this error and state that this does not change the scientific conclusions of the article in any way. The original article has been updated.

