# Supplementary material for: Review of public motor imagery and execution datasets in brain-computer interfaces
Source: Front Hum Neurosci. 2023 Mar 30;17:1134869. doi: 10.3389/fnhum.2023.1134869 (PMC10101208; doi:10.3389/fnhum.2023.1134869)
Supplement: Supplementary file 1 [file Data_Sheet_1.pdf]

Supplementary Material

Article Title Review of Public Motor Imagery and Execution Datasets in Brain-Computer Interfaces

Daeun Gwon, Kyungho Won, Minseok Song, Chang S. Nam, Sung Chan Jun and Minkyu Ahn*

*** Correspondence:**
Corresponding Author Minkyu Ahn
minkyuahn@handong.edu

1 Table of Definitions

We used a variety of terms in this study. Because they were often used as different words in papers, they have been defined as intuitively as possible (Table 1).

Table 1. List of word definitions

Word	Definitions
Block	Intervals that compose one trial in a paradigm such as pre-rest, imagination ready and imagination
Cue type	Form of visual stimulus used to deliver specific commands to subjects such as arrow, text, object, and figure
Event type	Non-numeric class name such as left and right hand
Explicit movement instruction (EMI)	Instructs a subject to imagine a precise, detailed action
Kinetic movement instruction (KMI)	Instructs a subject to imagine muscle movements or to perform kinetic motor imagery
Run	Experimental session performed for a day, although it has a rest interval of several minutes with respect to the subject's condition
Session	Experiment performed by a subject on different days with the same paradigm
Signal continuity	Whether the signal is continuous or a segmented signal according to the trial
Simple movement instruction (SMI)	Instruct a subject only on imaginary objects, left or right-hand, without detailed instructions on how to imagine them
Stimuli type	Stimulus to distinguish each experimental block, which includes a cue type

2 Difficulties and Cautions in Using Public Datasets

Here, we list certain things to consider when using datasets. These are the practical details we have gleaned by reviewing the public datasets collected. These details had been described largely in the resource, and can be known only empirically. There were even cases in which the contents in the paper differed from those in the actual data. We hope this section will help BCI researchers facilitate their data computing using public datasets.

Stieger et al. (2021) used 64 electrodes, and the actual data consist of 62 electrodes, but two of them are CB1 and CB2, which are not EEG electrodes, so they need to be removed during EEG analysis. Jeong et al. (2020) was released in a compressed 'gz' data format that needs sufficient specifications

beyond the data capacity of 226.29 gigabytes to decompress. Zhou (2020) consists of eight datasets recorded from 41 electrodes with a file name beginning with “A” and 12 datasets recorded from 26 electrodes with a file name starting beginning “S”. Wu (2020) lacks a data description, so it is ambiguous whether the corresponding paradigm is MI or ME. It consists of 10 runs in subjects 1 to 4, while subject 5 had only five runs.

Only eight of Kim et al. (2018)’s subjects had a different data structure from other subjects and used a ‘mat’ file format. Kaya et al. (2018) performed sessions one to three times per subjects for each paradigm, with significant differences in the number of trials per class (Table 4). The Cho et al. (2017) had a signal gain value of 32, and if researchers want to know the pure EEG power, they have to divide the signal‘s amplitude by 32. Lee et al. (2016) and Zhou et al. (2016) had markers for events, but no information was provided on the corresponding classes such as the left or right hand. Shin et al. (2017) stated that the sampling rate was 1000 Hz, but the actual signal was 200 Hz. Steyrl et al. (2016) explained in a reference paper that the sample consisted of 13 subjects, but the actual dataset consisted of 14 subjects.

Yi et al. (2014) provided no information about whether the data were preprocessed, but they had down-sampled to 200 Hz and segmented by trial. In addition, electrode information was not included, so we had to ask the author separately. Two institutions—Korea Research Institute of Standards and Science (KRISS) and Yonsei University—measured Ahn et al. (2013a) and each institution used a different data structure. The KRISS segmented the signal by the class and released the dataset without electrode information, and Yonsei University released the continuous raw signal. Grosse-Wentrup et al. (2009) has electrode information, but there are only numbers (coordinates), so their names should be inferred and used.

As EEG is sensitive to both the individual’s condition and the experimental environment, it can be assumed as an independent subject when measured on different days even for the same person. Stieger et al. (2021)conducted experiments with seven to 11 sessions for approximately eight weeks. Jeong et al. (2020) conducted three sessions at intervals of about a week, and Zhou(2020) ran seven sessions in two weeks. Lee et al. (2019) ran two sessions on different days, each of which consisted of 100 trials per class.

Kaya et al. (2018) had a large difference, not only in the number of trials but also in the date interval, and it was re-conducted with the same paradigm after at least two days to a maximum of approximately four months. Ofner et al. (2017) consisted of two sessions, and Zhou et al. (2016) conducted three sessions, several days to several months apart. Faller et al. (2012) performed five sessions on different days within a timeframe of five days, and Tangermann et al. (2012) performed measures on two different days. Leeb et al. (2007) conducted two sessions offline and three sessions online. By referring to this section, BCI researchers may reduce the trial and error when using these varied datasets.
